# High visceral fat with low subcutaneous fat accumulation as a determinant of atherosclerosis in patients with type 2 diabetes

**DOI:** 10.1186/s12933-015-0302-4

**Published:** 2015-10-07

**Authors:** Ryotaro Bouchi, Takato Takeuchi, Momoko Akihisa, Norihiko Ohara, Yujiro Nakano, Rie Nishitani, Masanori Murakami, Tatsuya Fukuda, Masamichi Fujita, Isao Minami, Hajime Izumiyama, Koshi Hashimoto, Takanobu Yoshimoto, Yoshihiro Ogawa

**Affiliations:** Department of Molecular Endocrinology and Metabolism, Graduate School of Medical and Dental Sciences, Tokyo Medical and Dental University, 1-5-45 Bunkyo-ku, Yushima, Tokyo, 113-8510 Japan; Center for Medical Welfare and Liaison Services, Tokyo Medical and Dental University, Tokyo, Japan; Department of Preemptive Medicine and Metabolism, Graduate School of Medical and Dental Sciences, Tokyo Medical and Dental University, Tokyo, Japan; CREST, Japan Agency for Medical Research and Development, Tokyo, Japan

**Keywords:** Visceral adiposity, Subcutaneous adiposity, Atherosclerosis

## Abstract

**Background:**

Abdominal visceral obesity has been reported to be associated with cardiovascular risks than body mass index, waist circumference, and abdominal subcutaneous fat. On the other hand, there is evidence that subcutaneous fat has a beneficial role against cardio-metabolic risks such as diabetes or dyslipidemia. However, little is known regarding the association between high visceral fat with low subcutaneous fat accumulation and the risk for atherosclerosis.

**Methods:**

This study was designed to elucidate whether high visceral fat with low subcutaneous fat accumulation enhances the risk for atherosclerosis in patients with type 2 diabetes. This is a cross-sectional study of 148 patients with type 2 diabetes (mean age 65 ± 12 years; 44.5 % female). Visceral fat area (VFA, cm^2^) and subcutaneous fat area (SFA, cm^2^) were assessed by abdominal computed tomography. Carotid intima media thickness (CIMT, mm) measured by ultrasonography was used for the assessment of atherosclerosis. Patients were divided into four groups: SFA < 100 cm^2^ and VFA < 100 cm^2^ [S(−)V(−)], SFA ≥ 100 cm^2^ and VFA < 100 cm^2^ [S(+)V(−)], SFA < 100 cm^2^ and VFA ≥ 100 cm^2^ [S(−)V(+)], and SFA ≥ 100 cm^2^ and VFA ≥ 100 cm^2^ [S(+)V(+)]. Linear regression analysis with a stepwise procedure was used for the statistical analyses.

**Results:**

Among the patients examined, 16.3 % were S(−)V(+). Mean (95 % confidence interval) of CIMT adjusting for age and gender were 0.80 (0.69–0.91), 0.86 (0.72–1.01), 1.28 (1.11–1.44) and 0.83 (0.77–0.88) in patients with S(−)V(−), S(+)V(−), S(−)V(+) and S(+)V(+), respectively (p < 0.001). The S(−)V(+) patients exhibited significantly older than S(−)V(−) patients and those with S(+)V(+) and had a highest VFA-SFA ratio (V/S ratio) among the four groups. S(−)V(+) patients were male predominant (100 % male), and S(+)V(−) patients showed female predominance (82 % female). In multivariate linear regression analysis (Adjusted R^2^ = 0.549), S(−)V(+) was significantly associated with CIMT (Standardized β 0.423, p < 0.001). Notably, S(+)V(+) was inversely associated with CIMT in the multivariate model.

**Conclusions:**

This study provides evidence that high visceral fat with low subcutaneous fat accumulation is an important determinant of carotid atherosclerosis and high subcutaneous fat could be protective against atherosclerosis in patients with type 2 diabetes.

## Background

Obesity has been reported to be associated with insulin resistance, dyslipidemia, and hypertension, thus increasing the risk for cardiovascular disease (CVD) [1–4]. Regarding body fat distribution, abdominal visceral fat has been more strongly associated with cardiovascular risks than body mass index (BMI), waist circumference, and abdominal subcutaneous fat [5, 6]. Therefore, evaluation and management of visceral fat accumulation is important to reduce cardio-metabolic burdens. Recently, we have reported that increased visceral fat with normal BMI is associated with arterial stiffening in patients with type 2 diabetes [7]. On the other hand, there is evidence that subcutaneous fat has a beneficial role against cardio-metabolic risks such as diabetes or dyslipidemia [8, 9]. These observations suggest the importance of direct evaluation of visceral and subcutaneous fat accumulation for the management of atherosclerosis; therefore it is possible that increased visceral fat with decreased subcutaneous fat accumulation is positively associated with atherosclerosis. Here we investigated the impact of body fat distribution, i.e. increased visceral fat with decreased subcutaneous fat accumulation, on carotid atherosclerosis in Japanese patients with type 2 diabetes.

## Methods

### Subjects

Patients with type 2 diabetes who regularly visited Tokyo Medical and Dental University Hospital participated in this study. Patients were eligible, if they were aged ≥20 years, and 148 consequential patients who underwent abdominal computed tomography (CT) for the assessment of visceral and subcutaneous fat accumulation were enrolled. Patients with severe renal impairment [estimated glomerular filtration rate (eGFR) <15 mL/min/1.73 m^2^ or undergoing renal replacement therapy], pregnant women, and those with infectious or malignant diseases were excluded. Type 2 diabetes was diagnosed according to the criteria of the Japan Diabetes Society (JDS) [10]. This study complies with the principles laid by Declaration of Helsinki and has been approved by the ethical committee of Tokyo Medical and Dental University (No. 2103).

### Clinical and biochemical analysis

Visceral fat area (VFA) and subcutaneous fat area (SFA) were measured at the level of umbilicus by abdominal CT examination (Aquilion PRIME, Toshiba Medical Systems, Tochigi, Japan). Atherosclerosis was assessed by carotid intima media thickness (CIMT) using an echotomographic system (Aplio XG SSA790A, Toshiba Medical Systems, Tochigi, Japan) with a 7.5-MHz linear transducer, as reported previously [11]. Following the criteria of visceral fat obesity as recommended by the Japan Society for the Study of Obesity [12], we defined visceral and subcutaneous fat accumulation; they were classified into four groups as follows: SFA < 100 cm^2^ and VFA < 100 cm^2^ [S(−)V(−)], SFA ≥ 100 cm^2^ and VFA < 100 cm^2^ [S(+)V(−)], SFA < 100 cm^2^ and VFA ≥ 100 cm^2^ [S(−)V(+)] and SFA ≥ 100 cm^2^ and VFA ≥ 100 cm^2^ [S(+)V(+)].

### Statistical analysis

Statistical analysis was performed using programs available in the SPSS version 21.0 statistical package (SPSS Inc., Chicago, IL, USA). Data are presented as mean ± SD or geometric mean with 95 % confidence interval (CI) as appropriate according to data distribution. Differences among the four groups were tested with a one-way ANOVA (continuous variables) or Chi square test (categorical variables) followed by Tukey–Kramer methods for the post hoc analyses. Linear regression analysis with a stepwise procedure was used to assess the cross-sectional association of each manifestation of abdominal (VFA) and subcutaneous (SFA) fat accumulation with carotid atherosclerosis. The following covariates were incorporated into the analysis; age, gender, duration of diabetes, smoking status, systolic blood pressure, triglycerides, high-density lipoprotein (HDL) cholesterol, low-density lipoprotein (LDL) cholesterol, HbA1c, urinary albumin-to-creatinine ratio (ACR), eGFR, and the use of insulin, the use of calcium channel blockers (CCBs), angiotensin-converting enzyme inhibitors, angiotensin receptor blockers (ARBs), statins, and anti-platelet agents. We also underwent a sensitivity analysis to examine the association of VFA and SFA with CIMT, using the cutoff of 100 and 150 cm^2^ in VFA and SFA, respectively, because the average of SFA in this study was approximately 150 cm^2^. Differences were considered to be statistically significant at p value less than 0.05.

## Results

**Fig. 1:**
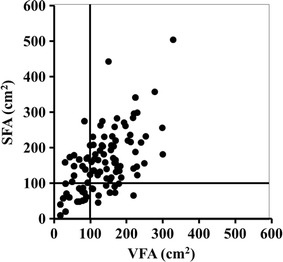
The correlation between visceral fat area and subcutaneous fat area in patients with type 2 diabetes. *SFA* subcutaneous fat area (cm^2^), *VFA* visceral fat area (cm^2^)

A total of 148 Japanese patients with type 2 diabetes (mean age 65 ± 12 years; 44.5 % female) were enrolled in this study. Among the participants, 16.3 % (N = 11) were classified as S(−)V(+), and 23.0 % (N = 34), 26.4 % (N = 39) and 43.2 % (N = 64) were classified as S(−)V(−), S(+)V(−), and S(+)V(+), respectively (Fig. 1). As shown in Table 1, S(−)V(+) patients were older than S(−)V(−) patients (p = 0.004) and S(+)V(+) (p = 0.036), and had a significantly higher VFA-SFA ratio (V/S ratio) than S(−)V(−) (p < 0.001), S(+)V(−) (p < 0.001), and S(+)V(+) patients (p < 0.001). Systolic blood pressure in S(−)V(+) patients were significantly higher than S(−)V(−) (p < 0.001) and S(+)V(−) patients (p < 0.001). Interestingly, there were appreciable differences in gender and fat distribution between S(−)V(+) and S(+)V(−) patients, without a significant difference in BMI. In this study, S(−)V(+) patients were male predominant (100 % male), and S(+)V(−) patients showed female predominance (82 % female). Moreover, S(−)V(+) patients had significantly higher uric acid (p < 0.001) and glutamyl transpeptidase (γ-GTP) (p < 0.001) levels than S(+)V(−) patients. The S(−)V(+) patients had reached the maximum BMI at younger age (43 ± 12 years) than S(+)V(−) patients (47 ± 11 years) and S(+)V(+) patients (51 ± 13 years). The maximum BMI in S(−)V(+) patients was significantly lower than that S(+)V(−) and S(+)V(+) patients (data not shown). Medications were listed in Table 2. None of S(−)V(+) patients received biguanides nor statins, although S(−)V(+) patients showed increased visceral fat accumulation. The number of S(+)V(+) patients taking biguanides and statins was greater than those of S(−)V(−), S(+)V(−), and S(+)V(+) patients.

**Table 1:** Clinical data of patients with type 2 diabetes

VFA (cm^2^)	<100		≥100		p value*
SFA (cm^2^)	<100 (N = 34)	≥100 (N = 39)	<100 (N = 11)	≥100 (N = 64)	
VFA (cm^2^)	56 ± 26	74 ± 21	149 ± 34	175 ± 52	<0.001
SFA (cm^2^)	61 ± 23	157 ± 41	78 ± 16	149 ± 34	<0.001
VFA-to-SFA ratio	1.00 ± 0.43	0.50 ± 0.20	1.98 ± 0.62	0.92 ± 0.33	<0.001
Age (years)	70 ± 10	64 ± 11	72 ± 6	62 ± 12	0.001
Gender (% male)	77	18	100	59	<0.001
BMI (kg/m^2^)	19.4 ± 2.1	23.2 ± 2.4	22.5 ± 1.8	27.0 ± 3.8	<0.001
SBP (mmHg)	118 ± 15	115 ± 9	136 ± 13	129 ± 11	<0.001
DBP (mmHg)	68 ± 11	66 ± 9	78 ± 15	75 ± 12	<0.001
Current smoker (%)	0	5	18	11	0.111
Duration of diabetes (years)	4.9 (4.2–5.7)	2.6 (2.2–3.2)	3.8 (3.0–4.8)	3.2 (3.1–3.3)	0.120
HbA1c (%)	6.9 ± 1.0	6.7 ± 0.4	6.7 ± 0.4	7.4 ± 1.7	0.024
Triglycerides (mmol/l)	1.36 (1.15–1.61)	1.01 (0.88–1.16)	1.16 (0.79–1.69)	1.67 (1.43–1.96)	0.001
HDL cholesterol (mmol/l)	1.48 ± 0.52	1.50 ± 0.58	1.61 ± 0.42	1.52 ± 0.44	0.900
LDL cholesterol (mmol/l)	2.42 ± 0.72	2.80 ± 1.05	2.80 ± 0.75	2.95 ± 0.82	0.052
Uric acid (μmol/l)	282 ± 85	265 ± 82	395 ± 91	335 ± 58	<0.001
eGFR (ml/min/1.73 m^2^)	76.8 ± 18.1	73.0 ± 21.5	62.3 ± 22.6	70.6 ± 23.4	0.250
Log ACR (mg/g)	24 (18–31)	23 (15–35)	25 (11–55)	45 (31–68)	<0.001
PDR (%)	6	0	0	11	0.128
AST (U/l)	25 (23–27)	21 (19–24)	27 (22–34)	25 (23–28)	0.060
ALT (U/l)	21 (17–25)	16 (14–17)	21 (14–31)	27 (23–31)	<0.001
γ-GTP (U/l)	26 (23–31)	23 (21–25)	64 (37–112)	41 (29–59)	<0.001
CIMT (mm)	0.86 ± 0.17	0.88 ± 0.07	1.30 0.41	0.81 ± 0.17	<0.001

Data are expressed as mean ± SD, geometric mean (95 % CI) or percentage

*ALT* alanine aminotransferase, *AST* asparatate aminotransferase, *CIMT* carotid intima media thickness, *DBP* diastolic blood pressure, *eGFR* estimated glomerular filtration rate, *γ-GTP* glutamyl transpeptidase, *HDL* high-density lipoprotein, *LDL* low-density lipoprotein, *PDR* proliferative diabetic retinopathy, *SBP* systolic blood pressure

* One-way ANOVA or Chi square test

**Table 2:** Medications of patients with type 2 diabetes

VFA (cm^2^)	<100		≥100		p value*
SFA (cm^2^)	<100 (N = 34)	≥100 (N = 39)	<100 (N = 11)	≥100 (N = 34)	
OHA (%)	41.2	33.3	54.5	54.7	0.164
Sulfonylureas (%)	25.0	0.0	12.5	22.4	0.055
Biguanides (%)	16.7	15.4	0.0	40.8	0.012
Alpha-GIs (%)	25.0	0.0	12.5	10.2	0.048
TZDs (%)	8.3	0.0	7.7	6.1	0.301
DPP4 inhibitors (%)	25.0	42.3	62.5	34.7	0.245
Glinides (%)	8.3	0.0	0.0	0.0	0.070
GLP-1 agonists (%)	0.0	0.0	0.0	2.0	0.754
Insulin (%)	41.2	38.5	18.2	35.9	0.575
ACEIs (%)	0.0	5.4	0.0	3.2	0.516
ARBs (%)	17.6	24.3	45.5	44.4	0.025
CCBs (%)	11.8	5.4	36.4	30.2	0.007
Beta blockers (%)	5.9	18.9	18.2	14.3	0.424
Alpha blockers (%)	0.0	5.4	0.0	1.6	0.383
Diuretics (%)	5.9	24.3	9.1	11.1	0.110
Statins (%)	11.8	16.2	0.0	34.9	0.007
Fibrates (%)	0.0	0.0	0.0	3.2	0.451
UA-lowering agents	0.0	10.8	9.1	6.3	0.289
Anti-platelets (%)	11.8	0.0	9.1	12.7	0.168

Data are expressed as percentage

*ACEI* angiotensin-converting enzyme inhibitor, *ARB* angiotensin receptor blocker, *CCB* calcium channel blocker, *DPP4* dipeptidyl peptidase-4, *GI* glycosidase inhibitor, *GLP-1* glucagon-like peptide**-**1, *OHA* oral hypoglycemic agent, *TZD* thiazolidinedione, *UA* uric acid

* Chi square test

As expected, S(−)V(+) patients had the highest CIMT level among the four groups [vs. S(−)V(−) (p < 0.001), vs. S(+)V(−) (p < 0.001), vs. S(+)V(+) (p < 0.001)] (Table 1). After adjustment for age and gender, mean (95 % CI) of CIMT were 0.80 (0.69–0.91), 0.86 (0.72–1.01), 1.28 (1.11–1.44) and 0.83 (0.77–0.88) in patients with S(−)V(−), S(+)V(−), S(−)V(+) and S(+)V(+), respectively (p < 0.001). On the other hand, CIMT level in S(+)V(+) patients was roughly equivalent to those in S(−)V(−) and S(+)V(−) patients. In the univariate analysis, S(−)V(+) was significantly associated with CIMT (standardized β 0.531, p < 0.001); whereas S(+)V(−) and S(+)V(+) were not associated with CIMT (Table 3). In the multivariate analysis, S(−)V(+) remained to be significantly associated with the risk for CIMT (standardized β 0.423, p < 0.001). Adjusted R^2^ was 0.549 in the model. Notably, S(+)V(+) was inversely associated with CIMT in the multivariate model. Using V/S ratio as the indicator for balance of visceral and subcutaneous fat accumulation, we also examined whether increased visceral fat relative to subcutaneous fat is continuously associated with CIMT. In this study, V/S ratio showed significantly positive correlations with CIMT in both univariate (Standardized β 0.506, p < 0.001) and multivariate linear regression analyses (Standardized β 0.383, p < 0.001). We finally underwent a sensitivity analysis using the cutoff of 150 cm^2^ for SFA because the average of SFA in this study was approximately 150 cm^2^. In the multivariate linear regression analysis, the association between SFA < 150 cm^2^ and VFA ≥ 100 cm^2^ and CIMT as compared with SFA < 150 cm^2^ and VFA < 100 cm^2^ reached a marginal statistical significance (Standardized β 0.190, p = 0.051); whereas, patients with SFA ≥ 150 cm^2^ and VFA ≥ 100 cm^2^ were not significantly increased risk for CIMT.

**Table 3:** Linear regression analysis for risk factors of intima media thickness in patients with type 2 diabetes

	Standardized β	p values
Univariates		
SFA ≥ 100 cm^2^ and VFA < 100 cm^2^ [S(+)V(−)]	0.032	0.813
SFA < 100 cm^2^ and VFA ≥ 100 cm^2^ [S(−)V(+)]	0.531	<0.001
SFA ≥ 100 cm^2^ and VFA ≥ 100 cm^2^ [S(+)V(+)]	−0.128	0.359
Multivariates		
SFA ≥ 100 cm^2^ and VFA < 100 cm^2^ [S(+)V(−)]	−0.100	0.386
SFA < 100 cm^2^ and VFA ≥ 100 cm^2^ [S(−)V(+)]	0.423	<0.001
SFA ≥ 100 cm^2^ and VFA ≥ 100 cm^2^ [S(+)V(+)]	−0.319	0.009
Age	0.575	<0.001
Urinary ACR	0.299	0.001
CCBs	−0.171	0.023
Duration of diabetes	0.156	0.049

Covariates; age, gender, history of cardiovascular disease, systolic blood pressure, duration of diabetes, current smoking, HbA1c, low-density lipoprotein cholesterol, high-density lipoprotein cholesterol, logarithmically transformed triglycerides, C-reactive protein, eGFR, albuminuria, the use of insulin, oral hypoglycemic agents, renin-angiotensin system blockers, calcium channel blockers and statins

*ACR* albumin-to-creatinine ratio, *CCB* calcium channel blocker, *SFA* subcutaneous fat area, *VFA* visceral fat area

## Discussion

Here, we demonstrate that S(−)V(+) patients are at an significantly increased risk for carotid atherosclerosis among Japanese patients with type 2 diabetes. Moreover, in multivariate analyses, there was a direct relationship between the presence of S(−)V(+) and risk for atherosclerosis and an inverse relationship between the presence of S(+)V(+) and risk for CIMT.

### The association between body fat accumulation and atherosclerosis

Visceral adipose tissue has been recently reported to be associated with coronary plaque characteristics in patients without diabetes [13] and visceral adipose tissue is a stronger risk factor of carotid atherosclerosis in Chinese adults [14]. Therefore, our data support the notion that visceral fat accumulation is positively associated with atherosclerosis. By contrast, Ravussin and Smith [15, 16] proposed the possibility that the ability to retain fat in subcutaneous depot is beneficial against cardio-metabolic risks. In addition, a more recent study clearly revealed that subcutaneous adipose thickness assessed by ultrasonography is inversely associated with carotid atherosclerosis in patients with type 2 diabetes [17]. These observations taken together, suggest that body fat distribution should be evaluated with information on visceral and subcutaneous fat accumulation for the assessment of the risks for atherosclerosis.

### Possible factors associated with fat distribution and atherosclerosis

In this study, S(−)V(+) patients were elderly men with severe cardio-metabolic profiles, including elevated blood pressure and uric acid, and high V/S ratio. These observations may partly explain the progression of atherosclerosis in S(−)V(+) patients. In addition, S(−)V(+) patients had reached maximum BMI at younger age than S(+)V(−) and S(+)V(+) patients. The maximum BMI in S(−)V(+) patients was low relative to S(+)V(−) and S(+)V(+) patients. It is interesting to speculate that S(−)V(+) patients have lower capacity to store excess energy in subcutaneous fat depot than S(+)V(−) and S(+)V(+) patients. Then, what could affect body fat distribution? A recent large scale cross-sectional study demonstrated that abdominal adiposity is positively associated with a deteriorated cardio-metabolic risk profile in multi-ethnicities and that East Asians have the highest visceral relative to subcutaneous fat accumulation among whites, African Caribbean blacks, Hispanics, East Asians, and Southeast Asians [18]. The Japanese men are likely to have a greater percent body fat than Australian men at any given BMI values [19]. Gender is also an important determinant of body fat distribution. Indeed, a genome-wide association study meta-analysis showed sexual dimorphism in the genetic regulation of fat distribution traits [20]. In this study, there was a clear gender difference in body fat distribution, with male predominance in S(−)V(+) and female predominance in S(+)V(−). It has been observed that high fat stores in ectopic fat compartments including skeletal muscle are present in male patients newly diagnosed with type 2 diabetes and altered lipid partitioning within muscle is independently associated with carotid atherosclerosis [21]. Després has recently proposed the lipid overflow-ectopic fat model [22]. If the extra energy is channeled into insulin-sensitive subcutaneous adipose tissue, the subjects will be protective against the development of the metabolic syndrome; whereas, in cases where the adipose tissue has a limited ability to store the excess energy into subcutaneous adipose tissue, triglycerides surplus will be deposited at undesirable sites such as skeletal muscle and visceral adipose tissue, leading the insulin resistance, atherogenic dyslipidemia, and atherosclerosis. Therefore, it is possible that low capacity of subcutaneous fat accumulation in patients with S(−)V(+) could allow ectopic fat accumulation within muscle as well as visceral fat accumulation, consequently leading to increased risk for carotid atherosclerosis. It remains to be determined whether the association observed between S(−)V(+) and atherosclerosis in Japanese subjects with type 2 diabetes will also be observed in other populations.

### Limitations

There are a couple of limitations in this study. First, it is impossible to infer causality because of its cross-sectional design. Second, we evaluated visceral fat and subcutaneous fat accumulation using VFA and SFA at the level of umbilicus; therefore, fat accumulation in other fat depots such as thighs and legs were not evaluated. Third, population in this study was ethnically and socially homogeneous, because this study was hospital-based; therefore, generalization of our findings might be limited. Fourth, we were unable to obtain information on diet and exercise in this study. These lifestyle could affect the distribution of body fat and BMI levels and could be one of the variables that is accounting for this high risk of CIMT in patients with S(−)V(+). Finally, it is important to undergo the sub-analyses to investigate the association of VFA and SFA accumulation with CIMT in different age groups, gender, and metabolic status; however, we could not undergo the analyses due to the relatively small sample size.

## Conclusions

It is of primary importance to identify diabetic patients who have advanced atherosclerosis because they are at extremely increased risk for CVD [23, 24]. Our data suggest that imbalance of visceral and subcutaneous fat distribution, i.e. increased visceral fat with decreased subcutaneous fat accumulation, is an important determinant of atherosclerosis, whereas increased subcutaneous fat accumulation could buffer the deleterious effect of visceral fat accumulation in patients with type 2 diabetes.

## Abbreviations

ACEI: angiotensin-converting enzyme inhibitor
ACR: albumin-to-creatinine ratio
ALT: alanine aminotransferase
ARB: angiotensin receptor blocker
AST: asparatate aminotransferase
BMI: body mass index
CCBs: calcium channel blockers
CI: confidence interval
CIMT: carotid intima media thickness
CT: computed tomography
CVD: cardiovascular disease
DBP: diastolic blood pressure
DPP4: dipeptidyl peptidase-4
eGFR: estimated glomerular filtration rate
GI: glycosidase inhibitor
GLP-1: glucagon-like peptide**-**1
GTP: glutamyl transpeptidase
HDL: high-density lipoprotein
JDS: Japan Diabetes Society
LDL: low-density lipoprotein
OHA: oral hypoglycemic agent
PDR: proliferative diabetic retinopathy
SBP: systolic blood pressure
SFA: subcutaneous fat area
TZD: thiazolidinedione
UA: uric acid
VFA: visceral fat area
